# Featureless Trachea Mimicking the Esophagus During Awake Fiberoptic Intubation Secondary to Longstanding Thyroid Compression

**DOI:** 10.7759/cureus.108757

**Published:** 2026-05-12

**Authors:** Keerthi Nadaraj, Mekha Elizabeth Mathew, Venkatesh Selvaraj

**Affiliations:** 1 Department of Anesthesiology, Sri Ramachandra Institute of Higher Education and Research, Chennai, IND

**Keywords:** awake fiberoptic intubation, difficult airway, thyroid mass, trachea, tracheomalacia

## Abstract

Giant thyroid masses causing prolonged tracheal compression may lead to severe tracheomalacia, where progressive erosion of cartilaginous rings renders the trachea smooth, featureless, and difficult to distinguish from the esophagus during bronchoscopy. This poses a significant risk during awake fiberoptic intubation. Such airway distortion requires heightened vigilance, meticulous endoscopic assessment, and confirmation of tracheal entry to prevent misplacement. Early recognition and a systematic airway strategy are crucial for optimal patient safety and outcomes.

## Introduction

Thyroid enlargement is a common endocrine disorder, and most thyroid swellings do not significantly compromise the airway. However, large thyroid masses (approximately a 20 x 10 cm mass in the anterior aspect of the neck) can lead to marked tracheal compression, deviation, and distortion of airway anatomy, making airway management during anesthesia particularly challenging. Long-standing thyroid enlargement may result in tracheal narrowing, tracheomalacia, or displacement of the trachea, which can significantly complicate endotracheal intubation and increase the risk of perioperative airway obstruction [1].

Securing the airway in such patients requires meticulous planning, as induction of general anesthesia in the presence of an anticipated difficult airway may result in loss of airway patency and inability to ventilate or intubate. Awake fiberoptic intubation is widely considered a preferred and well-established technique for the management of anticipated difficult airways, as it allows maintenance of spontaneous ventilation while enabling direct visualization of the airway structures [2].

In patients with large thyroid tumors, prolonged external compression may cause distortion or effacement of the tracheal cartilaginous rings, resulting in an atypical bronchoscopic appearance that can make identification of the trachea difficult [3]. Such unusual airway findings may create diagnostic uncertainty during fiberoptic intubation and require careful confirmation of airway anatomy.

We report a case of a large thyroid mass with severe tracheal distortion, in which the tracheal lumen appeared smooth and featureless during fiberoptic bronchoscopy, mimicking the esophagus, predominantly due to chronic mechanical compression of the thyroid leading to secondary physiological changes such as tracheal remodeling, mucosal apposition, and dynamic airway collapse, thereby posing a unique challenge to airway identification and management.

## Case presentation

**Figure 1:**
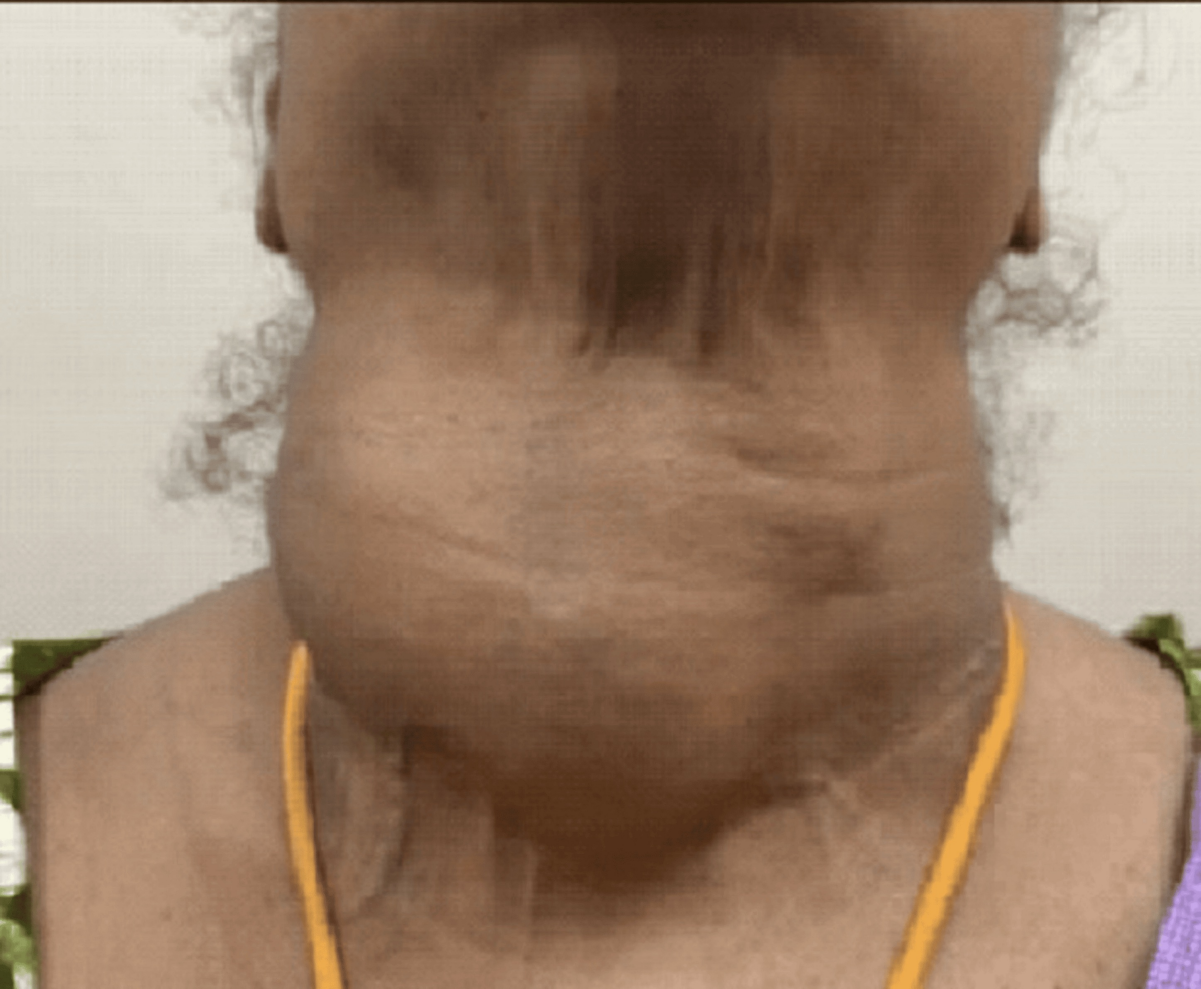
Thyroid mass Clinical photograph showing a giant anterior neck swelling (~20 x 10 cm) consistent with a longstanding thyroid mass.

A 60-year-old female presented with a 15-year history of progressively enlarging anterior neck swelling with recent hoarseness of voice and painful scalp swelling. She also had complaints of difficulty breathing on exertion and difficulty swallowing for the past three months. Examination revealed a large swelling of size 20 × 10 cm in the anterior aspect of the neck (Figure 1) that moves with deglutition but does not move on protrusion of the tongue, and no retrosternal extension was noted. Airway assessment showed Mallampati Grade IV with restricted neck extension.

**Figure 2:**
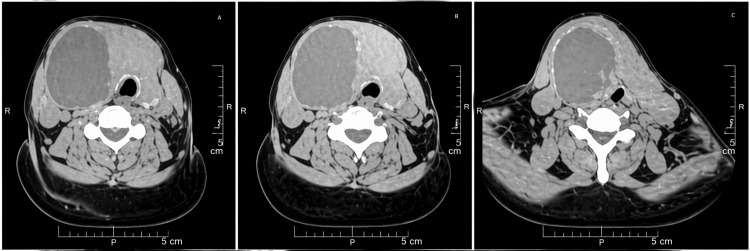
Computed tomography (CT) scan of the neck CT neck showing marked thyroid enlargement with tracheal compression and deviation, explaining the anticipated difficult airway. A: superior level showing thyroid mass compressing the trachea; B: midlevel showing enlarged thyroid with tracheal deviation; C: inferior level showing thyroid lobes and tracheal lumen narrowing.

**Figure 3:**
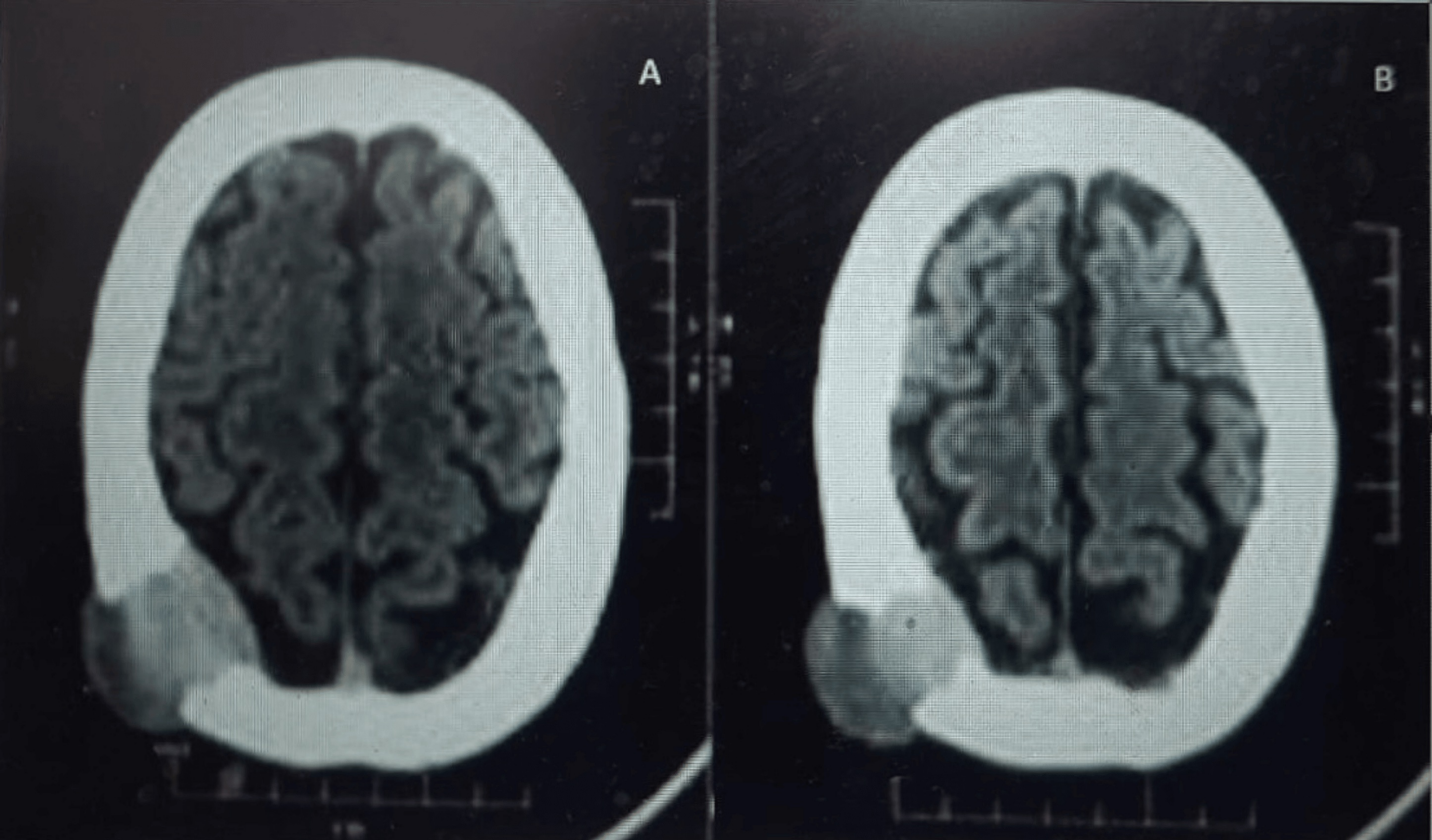
Computed tomography (CT) scan of the brain A, B: CT scan of the brain revealed a right parietal osseous lesion with calvarial destruction (which is located at approximately the 7 o’clock position in each image); the lesion was consistent with hematogenous metastasis from thyroid carcinoma.

CT neck confirmed diffuse thyroid enlargement (right lobe 7.2 × 5.7 × 9.0 cm; left lobe 5.6 × 2.1 × 4.2 cm) with significant tracheal deviation and mild luminal narrowing at the level of the thyroid gland (Figure 2). A CT brain revealed a right parietal osseous lesion with calvarial destruction, consistent with hematogenous metastasis from thyroid carcinoma (Figure 3).

In view of multiple predictors of a difficult airway, including Mallampati Grade IV, limited neck extension, reduced thyromental distance, and computed tomography evidence of tracheal deviation, awake fiberoptic intubation was selected as the primary airway management strategy for the planned total thyroidectomy.

The patient was counselled in detail regarding the rationale for awake intubation. Standard monitors were attached and intravenous access secured. Invasive blood pressure monitoring was done. After IV glycopyrrolate 0.2 mg premedication, conscious sedation was achieved with dexmedetomidine (1 µg/kg/min loading followed by 0.5 µg/kg/h maintenance); 2% lidocaine with adrenaline nasal pledgets, followed by 4% lidocaine nebulization, was used for airway topicalization. An adult fiberoptic bronchoscope preloaded with a 7.0 mm cuffed endotracheal tube was introduced orally; supraglottic structures, vocal cords, and glottis were clearly visualized and transited without difficulty.

**Figure 4:**
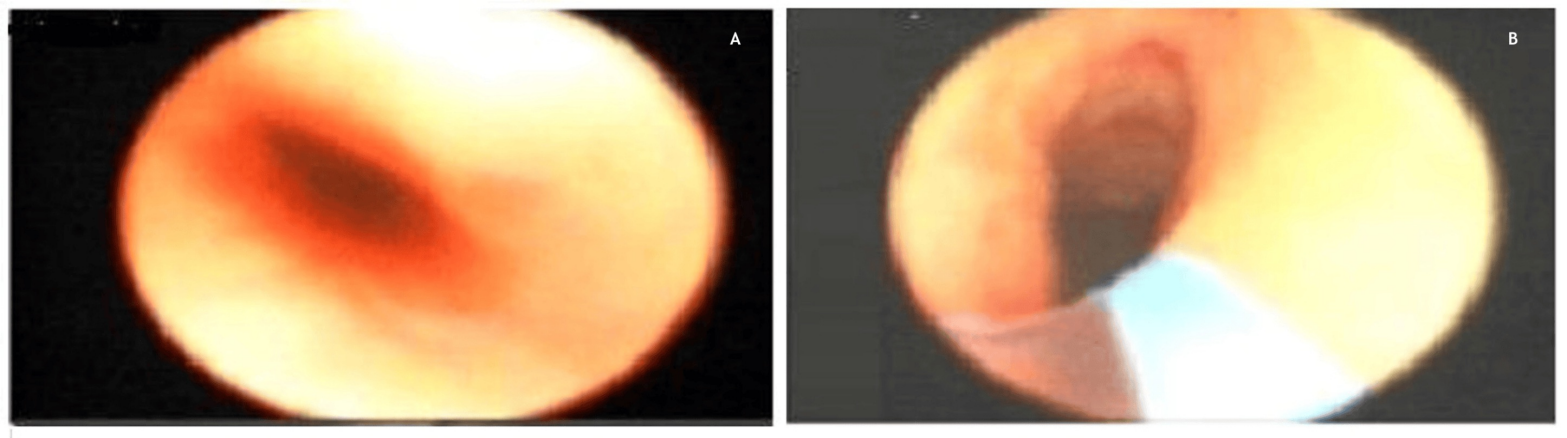
Fibreoptic bronchoscopic views of the tracheal lumen A: demonstrates the upper trachea with no cartilaginous rings, mimicking esophageal morphology; B: reveals the lower trachea with clearly identifiable tracheal rings.

Beyond the vocal cords, the tracheal lumen appeared smooth and featureless, devoid of cartilaginous rings, with pale, circumferentially uniform mucosa mimicking the esophagus, thereby raising immediate concern for inadvertent esophageal intubation (Figure 4). The bronchoscope was immediately withdrawn under continuous visualization, and laryngeal landmarks were promptly re-identified. The correct glottic passage was reconfirmed. Following anatomical reorientation, the scope was re-advanced cautiously. The smooth, featureless lumen was again encountered and traversed with strict midline orientation. Notably, fiberoptic bronchoscopy demonstrated dynamic inspiratory collapse of the upper trachea, localized to the segment with absent cartilaginous rings, consistent with tracheomalacia, whereas the distal trachea remained patent and structurally preserved.

Further advancement revealed distinct distal tracheal rings and a clearly visualized carina, confirming intratracheal placement. With the scope positioned in the mid-trachea, the 7.0 mm endotracheal tube was slowly railroaded. Placement was confirmed by bilateral equal air entry on auscultation and a sustained end-tidal CO₂ waveform, which is the definitive confirmatory modality when visual tracheal landmarks are unreliable. General anesthesia was induced with fentanyl (2 mcg/kg IV) and propofol (1.5 mg/kg IV) and maintained with sevoflurane. Neuromuscular blockade was achieved with vecuronium (0.1 mg/kg IV).

Following total thyroidectomy, intraoperative assessment revealed reduced tracheal rigidity involving approximately three to four upper tracheal rings. Given the risk of postoperative airway collapse, a joint decision by the surgical and anesthesiology teams was made to proceed with prophylactic tracheostomy for airway protection. Estimated blood loss was 800 ml, requiring one unit of packed red blood cell transfusion. Following reversal with intravenous neostigmine and glycopyrrolate, the patient was gradually weaned off from mechanical ventilation (Table 1).

**Table 1:** Clinical timeline BP: blood pressure; CT: computed tomography; ETCO₂: end-tidal carbon dioxide

Timeline	Clinical events
15 years prior	Gradual onset of anterior neck swelling, progressively increasing in size.
3 months prior to presentation	Development of dyspnea on exertion and dysphagia.
Recent weeks	Onset of hoarseness of voice and painful scalp swelling.
At presentation	Large anterior neck mass (~20 × 10 cm), moving with deglutition; Mallampati Grade IV, restricted neck extension.
Preoperative evaluation	CT neck: diffuse thyroid enlargement (right lobe 7.2 × 5.7 × 9.0 cm; left lobe 5.6 × 2.1 × 4.2 cm) with significant tracheal deviation and luminal narrowing. CT brain: right parietal osseous lesion with calvarial destruction, consistent with hematogenous metastasis from thyroid carcinoma.
Airway planning	Anticipated difficult airway due to multiple predictors → Awake fibreoptic intubation planned for total thyroidectomy.
Day of surgery (pre-induction)	Patient counseling; standard monitoring with invasive BP monitoring; airway topicalization (2% lidocaine with adrenaline nasal pledgets + 4% lidocaine nebulization); Conscious sedation with dexmedetomidine (1 μg/kg loading, then 0.5 μg/kg/h).
Airway management	Awake fibreoptic intubation performed. Beyond vocal cords, the tracheal lumen appeared smooth and featureless (esophagus-like) → scope withdrawn and laryngeal landmarks re-identified → correct glottic passage reconfirmed → scope re-advanced cautiously and traversed.
Bronchoscopic finding	Dynamic inspiratory airway collapse was observed in the upper trachea in the area lacking cartilaginous rings, consistent with tracheomalacia; the lower trachea remained patent without collapse.
Confirmation of placement	Visualization of distal tracheal rings and carina; placement confirmed by bilateral equal air entry on auscultation and sustained ETCO₂ waveform.
Intraoperative course	Following thyroidectomy, reduced tracheal rigidity involving 3–4 tracheal rings was noted.
Airway decision	Multidisciplinary decision → prophylactic tracheostomy performed at the end of the procedure to secure the postoperative airway.
Postoperative course	The patient was gradually weaned off from mechanical ventilation.

## Discussion

Giant thyroid masses may produce airway compromise ranging from asymptomatic tracheal deviation to life-threatening obstruction [1]. Chronic tracheal compression can progressively lead to deviation, luminal narrowing, and tracheomalacia resulting from loss of cartilaginous support [4]. Under normal conditions, the trachea is identified bronchoscopically by semi-circular cartilaginous rings with a posterior membranous trachealis muscle, whereas the esophagus appears smooth, pale, and circumferentially uniform without cartilaginous landmarks [5]. In advanced tracheomalacia, progressive erosion of the cartilaginous rings may produce a thin, highly compliant, featureless tracheal lumen that can be mistaken for esophageal mucosa during fiberoptic intubation, particularly in the presence of secretions or distorted anatomy [5,6].

Several features may aid differentiation even in advanced tracheomalacia. Scope trajectory may provide a clue, as the trachea generally follows a vertical course, whereas the esophagus deviates posteriorly. Visualization of the carina or tracheobronchial bifurcation remains the definitive anatomical confirmation of tracheal placement. Respiratory fogging may provide an additional clue, but it is not independently reliable. Continuous end-tidal CO₂ confirmation remains the single most definitive method of confirming tracheal intubation [7].

Post-decompression airway collapse remains a recognized concern in severe tracheomalacia and has traditionally prompted consideration of prophylactic tracheostomy [8]. However, serial fiberoptic bronchoscopy has also been proposed as a dynamic method to assess airway stability and help predict which patients may ultimately require tracheostomy versus conservative airway support [9]. This may be particularly valuable in patients with equivocal intraoperative findings. In this case, after thyroid gland excision, intraoperative findings demonstrated focal tracheal softening extending across approximately three to four tracheal rings, raising concern for underlying structural weakness. Based on these findings, a combined decision by the surgical and anesthesiology teams was made to proceed with tracheostomy at the end of the procedure.

Although tracheomalacia has historically been reported in patients with long-standing goiters, recent evidence suggests its true incidence may be substantially lower than traditionally assumed. In a large series, no cases of post-thyroidectomy tracheomalacia were identified, challenging the routine presumption of this complication in longstanding goiter. Similarly, a recent systematic review and meta-analysis confirmed tracheomalacia to be a rare event, with tracheostomy or prolonged intubation reserved for selected severe cases [10]. 

## Conclusions

This case demonstrates a rare bronchoscopic challenge in which advanced tracheomalacia due to a long-standing giant goiter leads to loss of normal cartilaginous landmarks, giving the trachea an esophagus-like appearance during awake fiberoptic intubation. Reliance on visual cues alone is unsafe; scope withdrawal to re-identify the glottis and cautious re-advancement with visualization of the entire length of the trachea, including the carina, is essential in cases of severe tracheomalacia to definitively confirm correct tracheal placement. Tracheal placement must be confirmed multimodally, with end-tidal CO₂ as the gold standard.
